# Adverse Effects Following Covishield and Covaxin Vaccination in Pregnant and Non-pregnant Women: A Comparative Study From Ranchi, Jharkhand

**DOI:** 10.7759/cureus.84112

**Published:** 2025-05-14

**Authors:** Indrani Dutta, Atima Bharti

**Affiliations:** 1 Obstetrics and Gynaecology, Manipal Tata Medical College, Jamshedpur, IND; 2 Obstetrics and Gynaecology, Rajendra Institute of Medical Sciences, Ranchi, IND

**Keywords:** aefi, covaxin, covid-19 vaccination, covishield, pregnancy

## Abstract

Background

Although COVID-19 vaccination in pregnancy has been approved by health authorities worldwide, data on adverse effects and safety profiles in pregnant women remain limited, particularly from diverse populations. Hence, this study aimed to compare the adverse effects following COVID-19 vaccination (Covishield and Covaxin) in pregnant and non-pregnant women in a tertiary care hospital in eastern India.

Methodology

This prospective, observational study was conducted at Rajendra Institute of Medical Sciences, Ranchi. Women aged 18-45 years receiving their first dose of COVID-19 vaccination were included and categorized into the following four groups: pregnant women receiving Covishield (Group A, n = 300), pregnant women receiving Covaxin (Group B, n = 357), non-pregnant women receiving Covishield (Group C, n = 446), and non-pregnant women receiving Covaxin (Group D, n = 100). Participants were followed up within 24 hours, at 7-14 days, after one month, and at delivery (for pregnant women). Adverse events following immunization (AEFIs) were recorded and analyzed using SPSS version 24.0 (IBM Corp., Armonk, NY, USA).

Results

Vaccine acceptance was 54.6% among pregnant women and 45.39% among non-pregnant women. The overall AEFI incidence rate was 41% for Covaxin and 24% for Covishield recipients (p < 0.001). Presence of allergy (adjusted odds ratio (AOR) = 1.950, 95% confidence interval (CI) = 1.379-2.757, p < 0.001) and vaccine type (AOR = 1.808, 95% CI = 1.408-2.322, p < 0.001) were significantly associated with AEFIs. Common adverse effects within 24 hours included fever, local pain at the injection site, and body aches, with similar profiles between pregnant and non-pregnant women. No significant adverse maternal or fetal outcomes were attributed to vaccination.

Conclusions

Both Covishield and Covaxin demonstrated acceptable safety profiles in pregnant and non-pregnant women, though Covaxin showed a higher AEFI rate. The types and frequencies of adverse effects were comparable between pregnant and non-pregnant women, suggesting that COVID-19 vaccination can be safely administered during pregnancy.

## Introduction

The COVID-19 pandemic has had a profound impact on global healthcare systems, with pregnant women particularly vulnerable to severe outcomes. Studies have demonstrated higher rates of pregnancy-induced hypertension, preeclampsia/eclampsia, and increased risk of admission to intensive care units (ICUs) among pregnant women with COVID-19 [1]. Nationwide lockdowns and anxiety about visiting medical facilities during the pandemic further compromised maternal and fetal well-being [2,3]. Evidence suggests that the pandemic significantly affected stillbirth and premature birth rates [4,5].

Recognizing the increased vulnerability of pregnant women to COVID-19 complications, the Ministry of Health and Family Welfare in India approved vaccination of pregnant women based on recommendations from the National Technical Advisory Group on Immunization [6]. According to these guidelines, pregnant women can be vaccinated at any time during pregnancy after receiving appropriate information about the risks, benefits, and potential side effects of vaccination.

In India, two main vaccines have been used for COVID-19 immunization, namely, the adenoviral vectored vaccine ChAdOx1 nCoV-19 (Covishield™, manufactured by the Serum Institute of India) and an inactivated whole virion vaccine BBV152 (Covaxin®, developed by Bharat Biotech). Pre-existing comorbidities, advanced maternal age, and high body mass index (BMI) have been identified as risk factors for severe COVID-19 outcomes in pregnancy [7]. Studies have demonstrated that pregnant women with symptomatic COVID-19 face increased risks of adverse pregnancy outcomes, including ICU admission [8-10].

Current evidence suggests that COVID-19 vaccines are safe during pregnancy and provide protection against COVID-19 [11,12]. Based on available knowledge, experts believe that COVID-19 vaccines are unlikely to pose significant risks to pregnant women or fetuses [12]. However, limited data exist regarding the adverse effects of vaccination in pregnancy across diverse populations, particularly concerning specific vaccine types, adverse effect profiles, and fetomaternal outcomes.

Given this knowledge gap, we conducted this study to compare the effects of COVID-19 vaccines (Covishield and Covaxin) in pregnant women with non-pregnant women of reproductive age (18-45 years) in a tertiary care hospital in eastern India. This study aims to provide valuable insights into vaccine safety profiles in this specific population and contribute to the growing body of evidence on COVID-19 vaccination during pregnancy.

## Materials and methods

Study design and setting

This prospective, observational study was conducted over six months at the Department of Obstetrics and Gynaecology and Vaccination Center of Rajendra Institute of Medical Sciences (RIMS), Ranchi, Jharkhand, India. Data collection was initiated after obtaining approval from the Institutional Ethics Committee of the concerned institution (approval number: 374, IEC, RIMS dated 08.10.2021).

Study population

The study included pregnant women attending antenatal checkups at the outpatient department and non-pregnant married women aged 18-45 years who visited the vaccination center for their first dose of COVID-19 vaccination. For sample size calculation, we referred to the phase 1 and 2 clinical trials of BBV152 (Covaxin) reported by Ella et al. [13], which showed that 6.1% of participants experienced systemic reactions after vaccination. We hypothesized that pregnant women, due to their immunocompromised status, might experience higher rates of systemic adverse effects (approximately 11%). To detect this clinically significant difference with 80% power and 5% alpha error (two-sided), we used the following formula for comparing two proportions:

n = [(Zα/2 + Zβ)² × {(p₁(1-p₁) + (p₂(1-p₂))}] ÷ (p₁-p₂)²

Where Zα/2 = 1.96 (for 95% confidence level), Zβ = 0.84 (for 80% power), p₁ = 0.11 (expected proportion of systemic reactions in pregnant women), and p₂ = 0.061 (proportion of systemic reactions from the Covaxin trials).

This yielded a minimum required sample size of 488 participants per vaccine group. Considering potential loss to follow-up in this longitudinal study, we added a 10% margin, resulting in a final target sample size of 1,084 participants (542 for Covishield and 542 for Covaxin). We further aimed to maintain a balanced distribution between pregnant and non-pregnant women in each vaccine group to enable appropriate comparisons.

Group allocation

Based on the availability of consenting participants taking either of the vaccines, they were sub-categorized into the following four groups: Group A (pregnant women who received Covishield, n = 300), Group B (pregnant women who received Covaxin, n = 357), Group C (non-pregnant women who received Covishield, n = 446), and Group D (non-pregnant women who received Covaxin, n = 100).

Covaxin is an inactivated vaccine, whereas Covishield is a viral vector vaccine. Following the guidelines of the Government of India, high-risk women (≥35 years, Class II obese and above, diabetic, hypertensive, those taking thyroid medication, and asthmatics on steroids and beta-agonists) were given Covaxin, while low-risk or no-risk individuals received Covishield.

Data collection

Study participants were assessed for adverse events following immunization (AEFIs) through telephonic conversations and during follow-up visits. Follow-ups were conducted within 24 hours of vaccination, 7-14 days post-vaccination, after one month, and at delivery (for pregnant women). A cause-effect relationship was established based on history, confirming no drug intake or other medical conditions immediately before or after vaccination. Comparisons were made between responses to Covishield and Covaxin in both pregnant and non-pregnant groups.

Data analysis

Data were analyzed using SPSS version 24.0 (IBM Corp., Armonk, NY, USA). Qualitative data were expressed as percentages and proportions, while quantitative data were presented as mean ± standard deviation. Univariate and binary logistic regression analyses were applied to determine associations between variables. A p-value of less than 0.05 was considered statistically significant.

## Results

Data from the hospital management information system of the hospital was accessed, which provided the records for 1,298 pregnant women and 5,429 non-pregnant women during the study period, of whom 709 pregnant women and 2,464 non-pregnant women were vaccinated against COVID-19. Overall, the vaccine acceptance rate was 54.6% among pregnant women and 45.39% among non-pregnant women. Table 1 presents the sociodemographic characteristics of the study participants. The majority of participants across all groups were above 30 years of age, except in Group D, where all participants were aged below 30 years. Educational status varied across groups, with graduates comprising a substantial proportion: 103 (34.3%) in Group A, 149 (41.7%) in Group B, 198 (44.4%) in Group C, and 51 (51.0%) in Group D. Occupation distribution showed diverse patterns, with government and private jobs being predominant. Most participants came from a low socioeconomic status.

**Table 1:** Sociodemographic characteristics of the participants.

Demographic features	Group A (n = 300), n (%)	Group B (n = 357), n (%)	Group C (n = 446), n (%)	Group D (n = 100), n (%)
Age				
<30 years	103 (34.3%)	159 (44.5%)	99 (22.2%)	100 (100%)
>30 years	197 (65.7%)	198 (55.5%)	347 (77.8%)	0 (0%)
Education				
Illiterate	49 (16.3%)	59 (16.5%)	47 (10.5%)	11 (11.0%)
Matriculation	53 (17.7%)	52 (14.6%)	78 (17.5%)	19 (19.0%)
Intermediate	95 (31.7%)	97 (27.2%)	123 (27.6%)	19 (19.0%)
Graduate	103 (34.3%)	149 (41.7%)	198 (44.4%)	51 (51.0%)
Occupation				
Housewife	52 (17.3%)	58 (16.2%)	48 (10.8%)	9 (9.0%)
Government job	97 (32.3%)	148 (41.5%)	122 (27.4%)	22 (22.0%)
Private	101 (33.7%)	101 (28.3%)	197 (44.2%)	49 (49.0%)
Business	50 (16.7%)	50 (14.0%)	79 (17.7%)	20 (20.0%)
Family size				
3–5 members	98 (32.7%)	154 (43.1%)	53 (11.9%)	47 (47.0%)
5–8 members	53 (17.7%)	99 (27.7%)	248 (55.6%)	33 (33.0%)
>8 members	149 (49.7%)	104 (29.1%)	145 (32.5%)	20 (20.0%)
Socioeconomic status				
Class I	151 (50.3%)	259 (72.5%)	243 (54.5%)	32 (32.0%)
Class II, III	98 (32.7%)	49 (13.7%)	153 (34.3%)	48 (48.0%)
Class IV, V	51 (17.0%)	49 (13.7%)	50 (11.2%)	20 (20.0%)
Residence				
Urban	151 (50.3%)	103 (28.9%)	197 (44.2%)	23 (23.0%)
Rural	149 (49.7%)	254 (71.1%)	249 (55.8%)	77 (77.0%)

Among pregnant women, the majority were multigravida: 257 (85.7%) in Group A and 323 (90.5%) in Group B. Among pregnant women, the majority were multigravida, with 257 (85.7%) in Group A and 323 (90.5%) in Group B. Most pregnant women were vaccinated between 25 and 30+6 weeks of gestation, followed by those at 36 weeks until delivery. Healthcare workers were the primary motivators for vaccination across all groups. The study included participants irrespective of prior history of COVID-19 infection. COVID-19 infection after vaccination was rare across all groups, affecting only 2-3% of participants in each group. Maternal and fetal outcomes showed similar patterns between Groups A and B, with no significant differences attributed to vaccine type (Table 2).

**Table 2:** Patient-specific characteristics. *: Percentages calculated among those who developed COVID-19 after vaccination. PPROM: preterm premature rupture of membranes; SGA: small for gestation age;; IUFD: intrauterine fetal demise; NICU: neonatal intensive care unit

Patient characteristics	Group A (n = 300), n (%)	Group B (n = 357), n (%)	Group C (n = 446), n (%)	Group D (n = 100), n (%)
Obstetrical history				
Primigravida	43 (14.3%)	34 (9.5%)	-	-
Multigravida	257 (85.7%)	323 (90.5%)	-	-
Gestational age			-	-
25–30+6 weeks	153 (51.0%)	167 (46.8%)	-	-
31–35+6 weeks	48 (16.0%)	47 (13.2%)	-	-
36 weeks till delivery	99 (33.0%)	143 (40.1%)	-	-
Motivator for vaccination				
Self	0 (0%)	0 (0%)	103 (23.1%)	12 (12.0%)
Family	11 (3.7%)	17 (4.8%)	99 (22.2%)	27 (27.0%)
Media	76 (25.3%)	101 (28.3%)	102 (22.9%)	30 (30.0%)
Health care worker	213 (71.0%)	239 (67.0%)	142 (31.8%)	31 (31.0%)
COVID-19 after vaccination				
Yes	3 (1.0%)	4 (1.1%)	2 (0.4%)	3 (3.0%)
No	297 (99.0%)	353 (98.9%)	444 (99.6%)	97 (97.0%)
Level of care after COVID-19 infection				
Home isolation	2 (66.7%)*	1 (25.0%)*	1 (50.0%)*	1 (33.3%)*
Hospitalization	1 (33.3%)*	3 (75.0%)*	1 (50.0%)*	2 (66.7%)*
Maternal outcomes (adverse if any)				
PPROM	27 (9.0%)	32 (9.0%)	-	-
Anemia	9 (3.0%)	8 (2.2%)	-	-
Hypertension	28 (9.3%)	33 (9.2%)	-	-
Gestational diabetes	31 (10.3%)	37 (10.4%)	-	-
Fetal outcome (adverse)				
SGA	3 (1.0%)	4 (1.1%)	-	-
IUFD	1 (0.3%)	2 (0.6%)	-	-
Preterm birth	27 (9.0%)	32 (9.0%)	-	-
NICU admission	8 (2.7%)	11 (3.1%)	-	-

Table 3 presents factors associated with AEFIs. AEFIs were reported in 189 (41%) Covaxin recipients and 178 (59%) Covishield recipients, with the differences being statistically significant (p < 0.001). Weight (<60 kg) and BMI (>25 kg/m^2^) were significantly associated with higher AEFI rates (p = 0.049 and p < 0.001, respectively). Individuals with allergies also experienced significantly higher rates of AEFIs (n = 166, 36.3% vs. n = 221, 29.7%, p = 0.009).

**Table 3:** Factors for adverse events following immunization among participants. ^#^: p-value was calculated using the chi-square test; *: P-values <0.05 were considered statistically significant. AEFI: adverse event following immunization; BMI: body mass index

Variable	Category	AEFI – yes, n (%)	AEFI – no, n (%)	P-value^#^
Type of vaccine	Covaxin	189 (41.0%)	272 (59.0%)	<0.001*
	Covishield	178 (24.0%)	564 (76.0%)	
Age	<30 years	146 (32.0%)	311 (68.0%)	0.132
	>30 years	209 (28.1%)	537 (71.9%)	
Ethnicity	Tribal	125 (27.4%)	332 (72.6%)	0.105
	Non-tribal	236 (31.7%)	510 (68.3%)	
Weight	<60 kg	151 (33.0%)	306 (67.0%)	0.049*
	>60 kg	210 (28.2%)	536 (71.8%)	
Height	<140 cm	143 (31.3%)	314 (68.7%)	0.239
	>140 cm	210 (28.2%)	536 (71.8%)	
BMI	<25 kg/m^2^	110 (24.1%)	347 (75.9%)	<0.001*
	>25 kg/m^2^	253 (34.0%)	493 (66.0%)	
Allergy	Yes	166 (36.3%)	291 (63.7%)	0.009*
	No	221 (29.7%)	525 (70.3%)	

**Figure 1:**
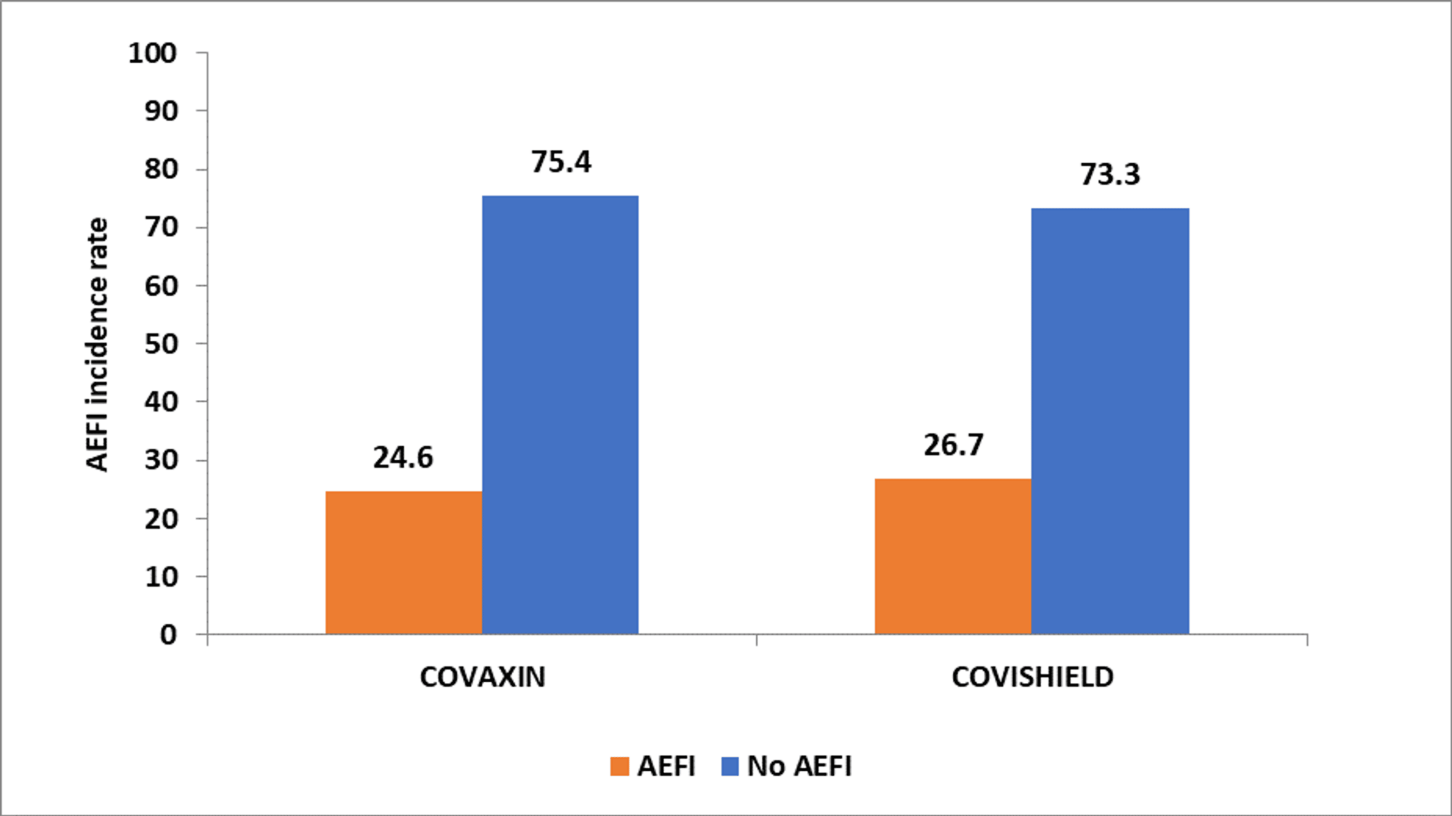
AEFI rates among pregnant women receiving Covaxin and Covishield. AEFI: adverse event following immunization

On stratified analysis, the AEFI rate was 26.7% (80 out of 300) among pregnant women receiving Covishield, and for pregnant women receiving Covaxin, the AEFI rate was 24.6% (88 out of 357), and the difference was not statistically significant (p = 0.555) (Figure 1).

Table 4 presents the results of binary logistic regression analysis for factors associated with AEFIs. After adjusting for potential confounders, the presence of allergy was found to be 1.950 times more likely to be associated with AEFIs (95% confidence interval (CI) = 1.379-2.757, p < 0.001). Similarly, vaccine type (Covishield vs. Covaxin) was 1.808 times more likely to be associated with AEFIs (95% CI = 1.408-2.322, p < 0.001).

**Table 4:** Bivariate logistic regression for factors associated with AEFI. *: P-values <0.05 were considered statistically significant. AOR: adjusted odds ratio; CI: confidence interval; BMI: body mass index; AEFI: adverse event following immunization

Variables	Category	AOR	95% CI (lower limit)	95% CI (upper limit)	P-value
Ethnicity	Non-tribal	0.986	0.760	1.280	0.918
	Tribal	Ref.	-	-	-
Weight	>60 kg	0.870	0.655	1.157	0.340
	<60 kg	Ref.	-	-	-
Height	>140 cm	0.744	0.529	1.046	0.089
	<140 cm	Ref.	-	-	-
BMI	>25 kg/m^2^	0.995	0.984	1.006	0.409
	<25 kg/m^2^	Ref.	-	-	-
Age	>30 years	1.049	0.804	1.368	0.724
	<30 years	Ref.	-	-	-
Allergy	Yes	1.950	1.379	2.757	<0.001*
	No	Ref.	-	-	-
Disease	Yes	1.017	0.714	1.449	0.926
	No	Ref.	-	-	-
Type of vaccine	Covishield	1.808	1.408	2.322	<0.001*
	Covaxin	Ref.	-	-	-

Table 5 presents the adverse effect profile observed within 24 hours of vaccination. Common adverse effects included fever, local pain at the injection site, and body aches, with similar frequencies across all groups. Fever and local pain at the injection site affected 80 (26.7%) participants in Groups A and B, 77 (17.3%) in Group C, and 72 (72.0%) in Group D. Body ache was reported by 76 (25.3%) participants in Group A, 68 (19.0%) in Group B, 66 (14.8%) in Group C, and 55 (55.0%) in Group D.

**Table 5:** Adverse effect profile in pregnant and non-pregnant vaccine recipients within 24 hours.

Adverse effect	Group A (n = 300), n (%)	Group B (n = 357), n (%)	Group C (n = 446), n (%)	Group D (n = 100), n (%)
Fever	80 (26.7%)	82 (23.0%)	77 (17.3%)	72 (72.0%)
Local pain at the injection site	80 (26.7%)	82 (23.0%)	77 (17.3%)	72 (72.0%)
Swelling at the injection site	7 (2.3%)	6 (1.7%)	6 (1.3%)	5 (5.0%)
Body ache	76 (25.3%)	68 (19.0%)	66 (14.8%)	55 (55.0%)
Sore throat	6 (2.0%)	5 (1.4%)	5 (1.1%)	4 (4.0%)
Cough	6 (2.0%)	5 (1.4%)	4 (0.9%)	4 (4.0%)

Table 6 shows the adverse effect profile 7-14 days post-vaccination. Pain in limbs was the most common side effect across all groups: 76 (25.3%) in Group A, 79 (22.1%) in Group B, 73 (16.4%) in Group C, and 70 (70.0%) in Group D. Persistent headache was also notable, particularly in 17 (4.8%) cases in Group B and 18 (18.0%) in Group D.

**Table 6:** Adverse effect profile in pregnant and non-pregnant vaccine recipients 7-14 days post-vaccination.

Adverse effect	Group A (n = 300), n (%)	Group B (n = 357), n (%)	Group C (n = 446), n (%)	Group D (n = 100), n (%)
Pain in limbs	76 (25.3%)	79 (22.1%)	73 (16.4%)	70 (70.0%)
Fever	7 (2.3%)	2 (0.6%)	1 (0.2%)	1 (1.0%)
Chest pain	1 (0.3%)	0 (0%)	1 (0.2%)	0 (0%)
Pain abdomen	1 (0.3%)	0 (0%)	1 (0.2%)	0 (0%)
Persistent headache	6 (2.0%)	17 (4.8%)	6 (1.3%)	18 (18.0%)
Blurring of vision	2 (0.7%)	0 (0%)	1 (0.2%)	0 (0%)
Pinpoint hemorrhage	0 (0%)	0 (0%)	0 (0%)	0 (0%)
Weakness of one side of the body	0 (0%)	0 (0%)	1 (0.2%)	0 (0%)
Seizures	1 (0.3%)	0 (0%)	0 (0%)	0 (0%)

No significant side effects were observed in any of the four groups after one month and at the delivery outcome.

## Discussion

This study aimed to compare the adverse effects of COVID-19 vaccines (Covishield and Covaxin) in pregnant and non-pregnant women in Ranchi, Jharkhand. Our findings provide important insights into vaccination acceptance, adverse event profiles, and associated factors in this population.

Vaccine acceptance

The COVID-19 vaccination uptake rate among pregnant women in our study was 54.6%, which is notably higher than rates reported in several other studies. Lipkind et al. [14] and Theiler et al. [15] reported lower COVID-19 vaccine uptakes of 21.8% and 7%, respectively, among pregnant women in the United States. Another study from North India by Gandhi et al. [16] showed a vaccination uptake rate of only 8% among pregnant women. Studies from the Czech Republic, France, and Ethiopia reported acceptance rates of 76.6%, 29.5%, and 62.04%, respectively, for COVID-19 vaccination among pregnant and postpartum women [17-19].

The higher acceptance rate in our study population may be attributed to several factors, including intensive awareness campaigns, strong motivation from healthcare workers (as evidenced by 67-71% of pregnant women citing healthcare workers as their primary motivator for vaccination), and the multi-country survey finding that vaccine acceptance for COVID-19 was generally the highest in India (up to 80%) [20]. This highlights the crucial role healthcare providers play in influencing vaccination decisions among pregnant women.

Demographic characteristics

In our study, 62.01% of participants were above 30 years old, which aligns with the findings from Levy et al. [21], who reported that most women accepting COVID-19 vaccination were over 30 years old (82.9%). Sutton et al. [22] found that the median age of pregnant women in their study was 36 years, further supporting our demographic profile.

Education played a significant role in vaccine acceptance, with participants having education up to intermediate (32.4%) or graduation (41.5%) forming the majority of vaccine recipients. This corresponds with findings from Levy et al. [21], who observed that pregnant women who accepted COVID-19 vaccines often had higher education levels, with 20.3% having high school degrees, 65% holding bachelor’s degrees, 61.6% with master’s degrees, and 66.3% with doctoral degrees.

Gestational age at vaccination

Our study found that most pregnant women were vaccinated between 25 and 30+6 weeks of gestation (48%). This differs somewhat from Lipkind et al. [14], who reported that the majority of vaccinated women were in their third trimester (61.8%). Gandhi et al. [16] and Gray et al. [23] found that the majority of vaccinated women were in their second trimester (45.3% and 46%, respectively). This variation may reflect different vaccination policies, healthcare provider recommendations, and participant concerns about vaccination timing during pregnancy.

Adverse events following immunization

The overall AEFI incidence rate in our study was 41% for Covaxin and 24% for Covishield recipients. This significant difference (p < 0.001) suggests that the type of vaccine is an important determinant of adverse events. Our logistic regression analysis confirmed that the vaccine type was significantly associated with AEFIs (adjusted odds ratio (AOR) = 1.808, 95% CI = 1.408-2.322, p < 0.001).

The presence of allergy was the strongest predictor of AEFIs (AOR = 1.950, 95% CI = 1.379-2.757, p < 0.001), emphasizing the importance of pre-vaccination screening for allergies. BMI >25 kg/m^2^ and weight <60 kg were also significantly associated with higher AEFI rates, which aligns with previous studies suggesting that body composition may influence immune responses to vaccination [24,25].

Adverse effect profile

The most common adverse effects within 24 hours of vaccination included fever, local pain at the injection site, and body aches, with similar frequencies across all groups. This is consistent with findings from Gandhi et al. [16], who reported fever (56.3%) and body pain (55.1%) as the most common AEFIs among pregnant women after 28 days. In contrast, studies from the United States reported local injection-site pain or soreness as the most common AEFI following the first dose (up to 88%), with only 1-4.2% reporting fever [23,24].

At 7-14 days post-vaccination, pain in limbs was the most common side effect across all groups, followed by persistent headache. These observations align with studies by Shimabukuro et al. [24] and Kadali et al. [25], who reported similar adverse effect profiles. Notably, the overall 28-day incidence of AEFIs in pregnant women did not differ significantly from that in non-pregnant women, consistent with the findings of these studies.

More severe adverse events, such as weakness on one side of the body and seizures, were rare, occurring in only one case each. No thromboembolic events were reported in our study population, which aligns with the generally favorable safety profile reported in larger studies [26].

Maternal and fetal outcomes

Our study found no significant adverse maternal or fetal outcomes attributed to vaccination. The rates of preeclampsia, gestational diabetes, preterm birth, and neonatal intensive care unit admissions were comparable between the Covishield and Covaxin groups, suggesting that the vaccine type does not significantly impact pregnancy outcomes. Goldshtein et al. [26] reported a median duration of AEFIs among pregnant women to be one day, which is consistent with our observation that most adverse effects manifested within 24 hours and resolved quickly.

No abortions, stillbirths, or intrauterine deaths directly attributable to vaccination were reported in our study, further supporting the safety of COVID-19 vaccination during pregnancy. This aligns with findings from multiple studies that have found no increased risk of adverse pregnancy outcomes following COVID-19 vaccination [14,16,23,26].

Strengths and limitations

The greatest strength of our study is the large number of participants, which allowed for enhanced reliability of the study findings. However, the study has several limitations. First, the allocation of Covaxin and Covishield was based on risk status rather than randomization, which may have introduced selection bias. High-risk women were given Covaxin, while low-risk women received Covishield, potentially confounding the association between vaccine type and AEFIs. Second, our study included only third-trimester pregnant women, limiting the generalizability of our findings to women in earlier stages of pregnancy. Third, the follow-up period was relatively short, limiting our ability to detect long-term effects of vaccination. Finally, the retrospective nature of AEFI assessment through telephonic interviews may have introduced recall bias.

## Conclusions

The COVID-19 vaccination uptake rate was 54.6% among pregnant women and 45.39% among non-pregnant women in our study population. The AEFI overall incidence rate was 40.9% for Covaxin and 24% for Covishield recipients, with the presence of allergy and vaccine type being significantly associated with AEFI. Common adverse effects within 24 hours included fever, local pain at the injection site, and body aches, with similar profiles between pregnant and non-pregnant women. No significant difference in AEFI was found between pregnant and non-pregnant women, suggesting that pregnancy does not substantially alter the safety profile of COVID-19 vaccines. No severe adverse events or poor pregnancy outcomes attributable to vaccination were observed, supporting the safety of COVID-19 vaccination during pregnancy. Our findings contribute to the growing body of evidence regarding the safety of COVID-19 vaccination in pregnant women, particularly in the Indian context. These results may help inform vaccination policies and address vaccine hesitancy among pregnant women. Future studies with randomized designs, larger sample sizes, and longer follow-up periods are recommended to further evaluate the long-term safety and efficacy of COVID-19 vaccines in this population.
